# Oral minoxidil treatment for hypotrichosis in Lelis syndrome

**DOI:** 10.1016/j.jdcr.2025.04.039

**Published:** 2025-05-29

**Authors:** Ishan Bhanot, Anuj Kunadia, Anisha Bhanot, Amanda S. Weissman, Benjamin Levin, Jeffrey McBride, Jarad Levin

**Affiliations:** a College of Medicine, University of Oklahoma Health Sciences Center, Oklahoma City, Oklahoma; b Department of Dermatology, University of Oklahoma Health Sciences Center, Oklahoma City, Oklahoma

**Keywords:** alopecia treatment, Ectodermal Dysplasia-Acanthosis Nigricans syndrome, hair regrowth, hypohidrosis, hypotrichosis, Lelis syndrome, oral minoxidil

## Introduction

Ectodermal dysplasias are a group of inherited disorders characterized by alterations in 2 or more ectodermal structures, usually affecting the hair, teeth, nails, and/or sweat glands. A review of ectodermal dysplasias identified more than 180 variants, including Lelis syndrome, a rare condition characterized by acanthosis nigricans, hypohidrosis, and hypotrichosis.^1^^,^^2^ Patients with Lelis syndrome may also present with hyperconvex, dystrophic nails, absent lower eyelashes, absent pubic and axillary hair, and facial dysmorphia with a long, narrow face, and upslanting palpebral fissures.^3^ The classical findings of hypohidrosis and hypotrichosis are key aspects for making the diagnosis and present therapeutic challenges.^2^

Hypotrichosis in the context of Lelis syndrome results from the aberrant development of hair follicles, a symptom that profoundly affects the patient's quality of life. As there is currently no cure for Lelis syndrome, treatment primarily focuses on alleviating symptoms.^2^ Yoshimura et al reported that treatment with acitretin improved the hyperkeratosis, acanthosis nigricans, and facial comedones of a 31-year old diagnosed with Lelis syndrome.^4^ However, the patient’s hypotrichosis remained unaffected with this treatment regimen.

## Report of a case

**Fig 1:**
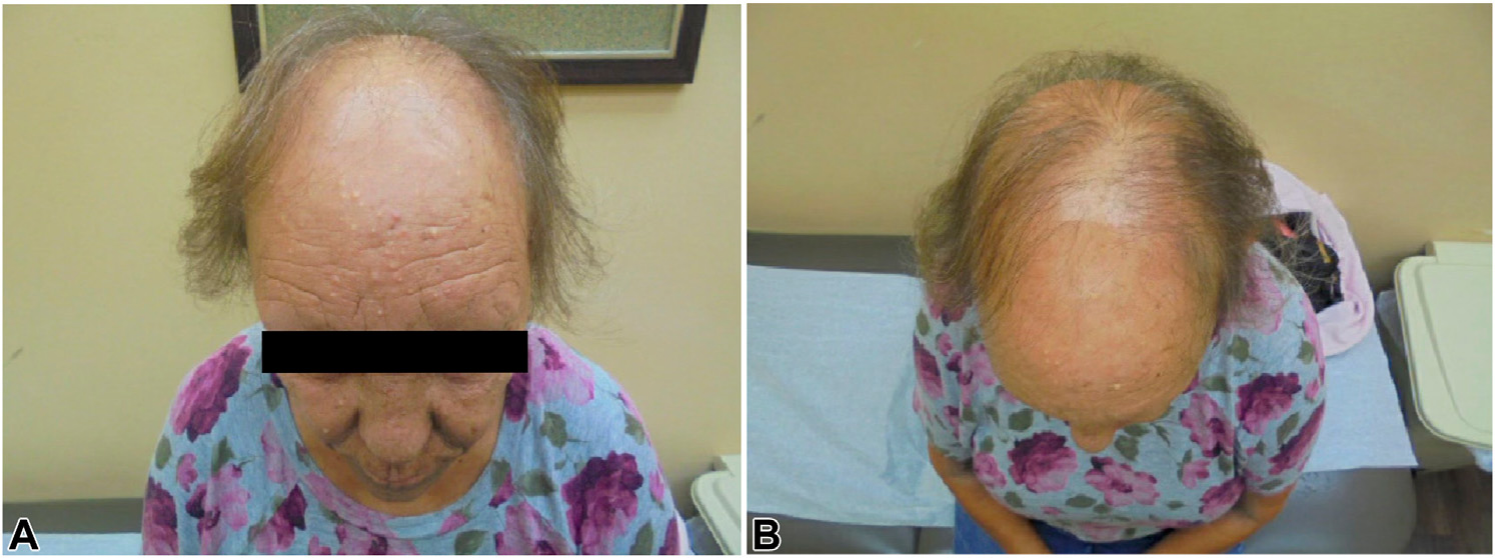
**A** and **B,** Pretreatment images showing diffuse hair thinning across the frontal and vertex scalp.

**Fig 2:**
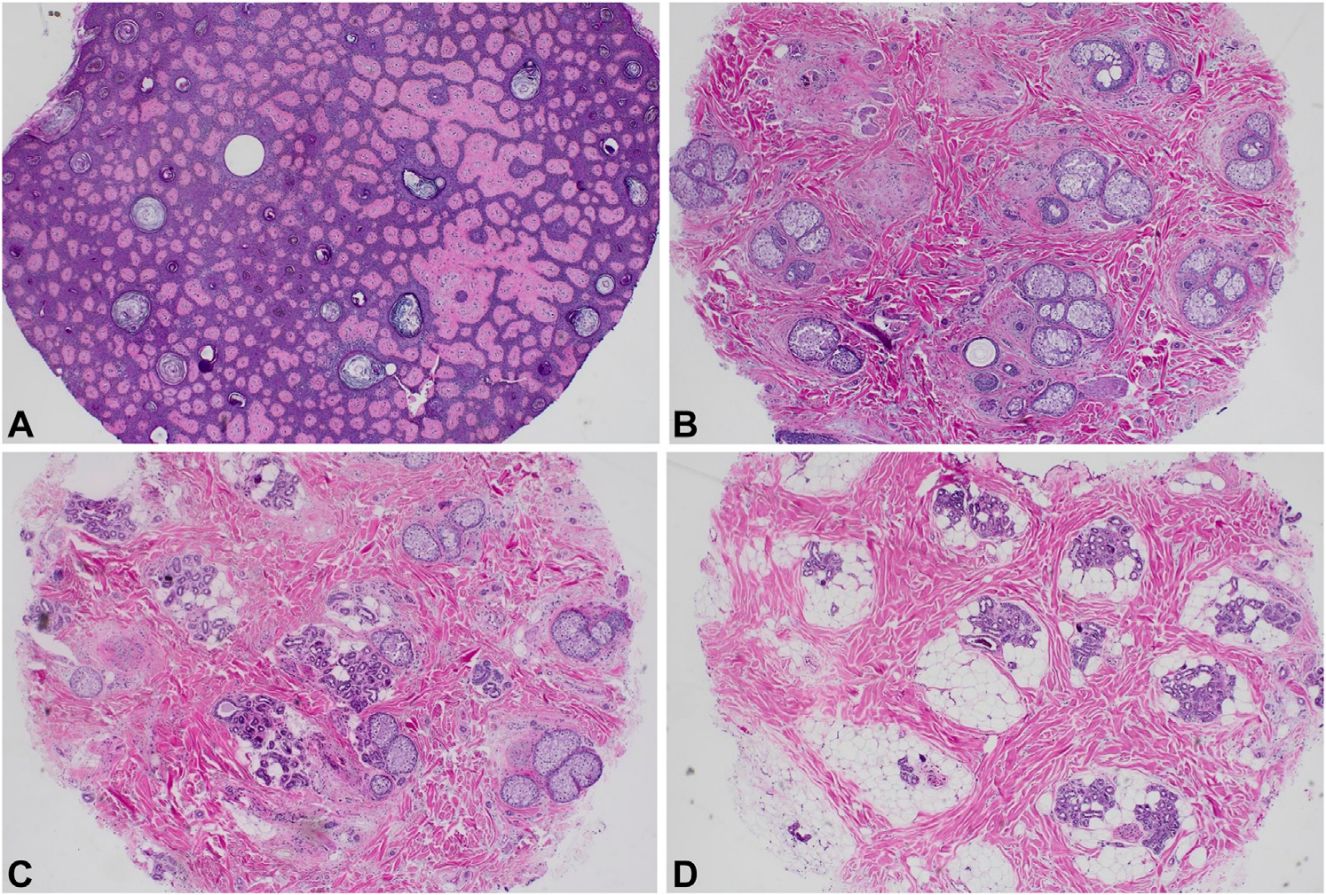
Alopecia in Lelis syndrome. **A,** Horizontal sections at epidermal levels reveal numerous milia-like cysts. Magnification: **(A)** = 40×. **B** and **C,** Horizontal sections at superficial dermal levels reveal preservation of sebaceous glands, eccrine glands, and miniaturized follicles. Some areas appears to lack normal pilosebaceous units. Magnification: **(B)** = 40×; **(C)** = 40×. **D,** Horizontal sections at the lower dermis and subcutaneous tissue reveal preservation of eccrine glands and a significant decreased density of in anagen hair follicles. Magnification: **(D)** = 40×.

**Fig 3:**
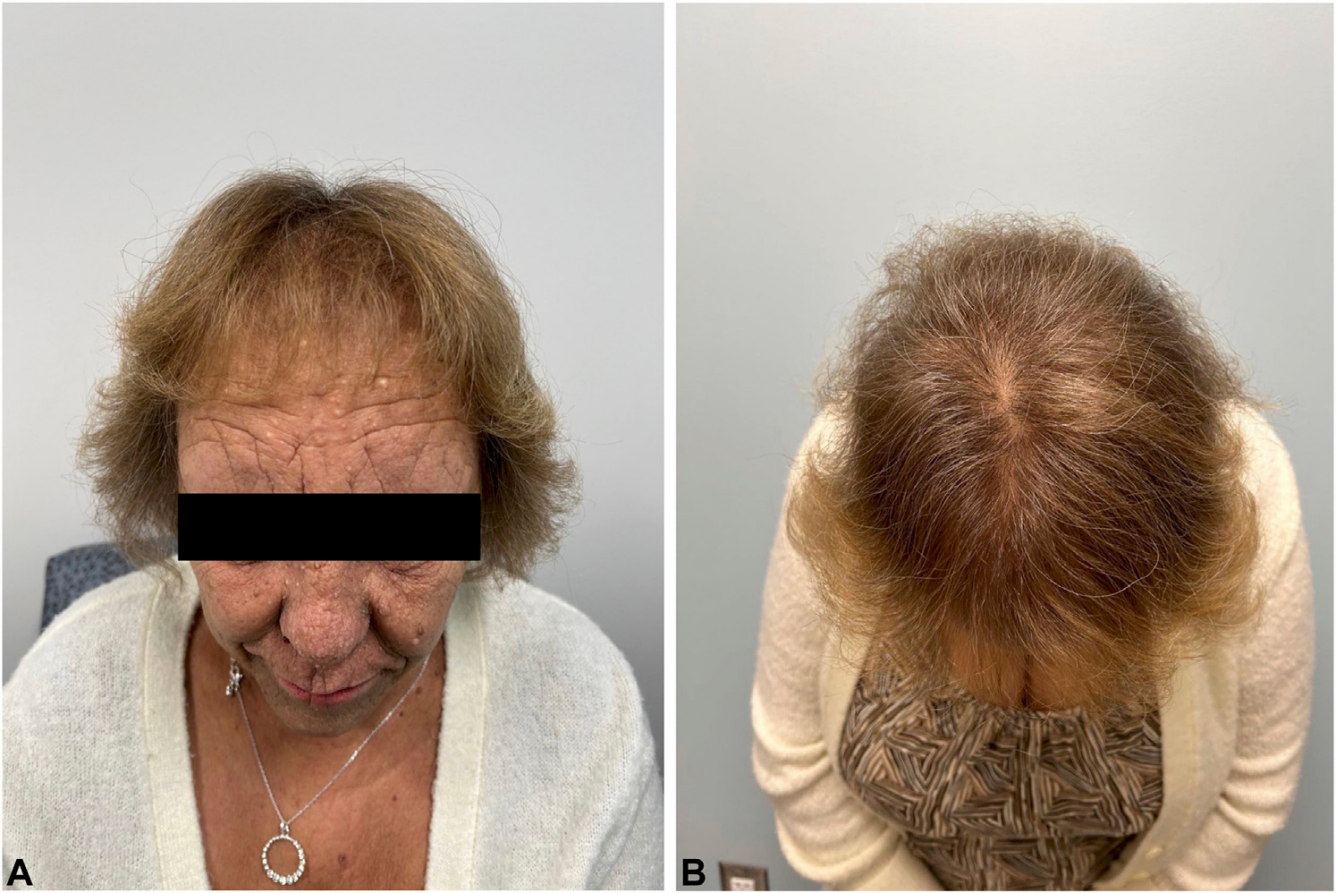
**A** and **B,** Significant hair growth and increased density observed after 5 months of oral minoxidil treatment (1.25 mg daily).

A female in her 60s with Lelis syndrome manifested by hypohidrosis and acanthosis nigricans presented to our clinic (University of Oklahoma; Oklahoma City) in 2022 for evaluation of progressive hair loss, with evidence of diffuse hair thinning of the frontal and vertex scalp (Fig 1, *A* and *B*). A scalp biopsy showed significant decrease in the number of regular terminal anagen follicles with miniaturized hair follicles and preservation of sebaceous and eccrine density and lack of inflammation. There was evidence of milia-like cysts which may relate to the dysregulated epidermal development and follicular maintenance in patients with Lelis syndrome (Fig 2). The patient was initiated on an oral minoxidil treatment regimen starting at 1.25 mg daily. After observing positive hair regrowth at her 5 month follow up, the dose was increased to 2.5 mg daily. Continuous treatment with this increased dose has resulted in sustained and significant improvement in hair density without any reported systemic adverse effects (Fig 3, *A* and *B*).

## Discussion

Minoxidil, a vasodilator medication traditionally used to treat androgenetic alopecia, has shown potential in promoting hair growth. Although the mechanism of action is not fully understood, minoxidil has been found to prolong the anagen phase of dermal papilla, increase VEGF mRNA expression, and stimulate prostaglandin E_2_ production, all of which induce hair growth.^5^ Topical minoxidil has been utilized off-label to treat many types of alopecia, including cases of hypohidrotic ectodermal dysplasia.^6^ While topical minoxidil has demonstrated efficacy, many patients are poorly compliant due to the appearance, cost, and side effects such as contact dermatitis, headaches, hair shaft brittleness, and hypertrichosis in the applied areas. Instead, oral minoxidil has shown to be a successful alternative option for this subset of patients with hair loss.^5^ Given the evidence of oral minoxidil’s effectiveness in inducing hair growth in similar dermatological conditions to Lelis syndrome, its application was utilized in the treatment of our patient.

## Conclusion

To our knowledge, medical literature regarding the use of oral minoxidil to treat hypotrichosis in the setting of Lelis syndrome is rare. Existing medical literature describes the use of topical minoxidil to address congenital alopecia in cases of hypohidrotic ectodermal dysplasia. However, topical minoxidil may have poor patient adherence due to the above-mentioned concerns of cosmesis, cost, and side-effects, and oral minoxidil is an effective alternative option. This case underscores the potential of low-dose oral minoxidil as a viable treatment option for alopecia in patients with ectodermal dysplasia, warranting further investigation into its long-term efficacy and safety profile.

Existing medical literature supports the use of topical minoxidil to treat alopecia in hypohidrotic ectodermal dysplasia, but the literature is sparse regarding the use of oral minoxdil. This case underscores the potential of low-dose oral minoxidil as a viable, safe, long-term treatment alternative for alopecia, not only in Lelis syndrome and other forms of ectodermal dysplasia, but in numerous other syndromes associated with noninflammatory alopecia.

## Conflicts of interest

None disclosed.
